# Preprint policies across journals and publishers in ecology and evolutionary biology

**DOI:** 10.1098/rspb.2025.0524

**Published:** 2025-11-12

**Authors:** Marija Purgar, Edward R. Ivimey-Cook, Antica Culina, Joshua D. Wallach

**Affiliations:** ^1^ Ruđer Bošković Institute, Zagreb 10000, Croatia; ^2^ Department of Epidemiology, Rollins School of Public Health, Emory University, Atlanta, GA 30322, USA; ^3^ University of East Anglia, Norwich NR4 7TJ, UK

**Keywords:** ecology, evolutionary, biology, journals, publishers, preprints, policies, instructions, recommendations

## Abstract

Preprints have the potential to accelerate knowledge dissemination and promote transparency in ecology and evolutionary biology. However, concerns about journal policies regarding prior publication may discourage researchers from preprinting their manuscripts. Therefore, we identified 230 eligible ecology and evolutionary biology journals, published by 69 different publishers, and assessed both their journal- and publisher-level preprint policies. At the journal level, 119 (51.7%) of the 230 journals included preprint policies in their author guidelines—either through journal-specific policies (109, 47.4%) or by directly referencing their publisher’s preprint policies (10, 4.3%). Overall, 116 (97.5%) of these journals were supportive of considering preprints for publication. At the publisher level, 26 (37.7%) of the 69 publishers had explicit preprint policies, all of which supported considering preprints for publication. There were 38 (16.5%) journals without journal- or publisher-level preprint policies. While most journals and publishers were supportive of considering preprints for publication, instructions for authors, such as acceptable locations for posting preprints, timing of preprint posting relative to manuscript submission and requirements to link preprints to final published articles, were lacking. These findings highlight opportunities for ecology and evolutionary biology journals, along with their publishers, to clarify and refine their preprint policies and instructions for authors.

## 1. Introduction

Timely access to scientific results in ecology and evolutionary biology is essential for shaping effective policies and management strategies related to conservation efforts [1–4] and climate change mitigation [4–6]. However, ecological studies often face significant publication delays [7], and their results may not be accessible due to journal paywalls [8,9], potentially limiting their impact. For instance, it has been estimated that nearly 50% of ecological research remains unpublished [10]. Preprints, which are preliminary research reports that have not been peer-reviewed [11], offer a potential solution to these challenges [12, 13].

Preprints play a crucial role in establishing the provenance of ideas [11,14,15], promoting transparency by making hard-to-publish studies openly available [11,14], accelerating the dissemination of findings [11,14,16] and fostering community feedback [17,18]. Preprints are especially beneficial for early-career researchers [19,20] who often face challenges in building publication records to secure funding, scholarships, grants or faculty positions [19]. Posting preprints is a well-established practice in fields such as physics [21], mathematics [21] and economics [22], and recent studies [23,24] suggest there is increasing interest in this practice within ecology and evolutionary biology. However, researchers often worry that posting a preprint before submitting to a peer-reviewed journal could conflict with a journal’s policy on prior publication [25]. Journals and publishers establish these policies to define manuscript eligibility and specify their stance on preprints, but the absence of clear instructions may make authors reluctant to preprint their manuscripts.

Previous studies have evaluated the preprint policies of journals across several fields [26,27]. A 2020 analysis [26] of the top 100 journals in clinical medicine found that while most journals allowed preprints, 14 either evaluated them on a case-by-case basis—assessing whether a submitted manuscript provided meaningful new insights or duplicated existing preprint content—or explicitly prohibited preprints altogether. According to a study [27] on preprint policies from journals across other scientific fields, over one-third provided unclear information on whether preprints could be posted online prior to manuscript submission. In a 2018 analysis [28] of preprint policies from 14 academic publishers, approximately 80% had a preprint policy. This study utilized Open Policy Finder (formerly Sherpa Romeo), an online resource that provides publisher-level open access archiving and compliance policies. However, little is known about the extent to which ecology and evolutionary biology journals, as well as their publishers, have established preprint policies that are supportive of considering preprints for publication and provide clear instructions for authors, such as acceptable locations for posting preprints and requirements regarding the timing of posting preprints relative to manuscript submission.

Therefore, we systematically assessed preprint policies across 230 ecology and evolutionary biology journals and their publishers to evaluate the current state of preprint policies and to inform potential changes that could help standardize practices across journals and publishers, clarify instructions for authors and promote the broader adoption of preprints in these fields.

## 2. Material and methods

We used the Web of Science 2023 Journal Citation Reports (JCR) filter with the categories 'Ecology' (195 journals) and 'Evolutionary Biology' (54 journals) to identify a list of journals that publish ecology and evolutionary biology research. There were 18 journals included in both categories. We excluded multidisciplinary journals, such as *Science* or *Nature*, as they encompass research across a range of scientific disciplines, and journals that were inactive due to upcoming publisher transitions (e.g. *Neotropical Biodiversity*). In total, we identified 230 eligible ecology and evolutionary biology journals (electronic supplementary material, table S1). Relevant journal-level metadata was retrieved from the Web of Science 2023 JCR list, including total citations, 2023 Journal Impact Factor (JIF) and the percentage of Open Access (OA) Gold content from journals with an OA option. These metrics were used to contextualize the visibility and publishing characteristics of journals in our sample.

Two authors (E.I.C. and M.P.) reviewed each journal’s website to identify specific preprint policies, including instructions regarding the possible locations for posting preprints (e.g. on a preprint server), disclosure of preprints at the time of manuscript submission (e.g. in a cover letter), the timing of preprint posting relative to manuscript submission (e.g. before submission to a journal) and any additional actions upon publication of the final article (e.g. requirements to link the preprint to the final published article). The authors also reviewed the preprint policies and related instructions of the corresponding publishers. For journals and publishers without clearly described preprint policies, we checked the Open policy finder (https://openpolicyfinder.jisc.ac.uk/search) and the first 10 pages of a Google search combining the journal name and the word ‘preprint’.

The journal- and publisher-level policies were classified using previously established categories [26]: manuscripts with preprints allowed without any restrictions, case-by-case determination (e.g. manuscripts with preprints were evaluated by the journal on an individual basis) or preprints prohibited (manuscripts with preprints were not considered for publication). If no preprint policy was identified at either the journal or publisher level, the journal was classified as having no preprint policy.

To assess whether preprint polices vary across JIF, we used a Wilcoxon rank-sum test to compare median 2023 JIF between journals with a preprint policy (either journal level or publisher level) and those without any preprint policy.

All data were collected between 27 November and 11 December 2024, and we posted our protocol on Open Science Framework (https://osf.io/tpu2q/overview) prior to data collection. Descriptive analyses were conducted using R v.4.4.2 [29]. The data tidying, filtering and graphing were performed using packages from the ‘Tidyverse’ version 2.0.0 [30]. More details on data processing are available in the electronic supplementary material.

## 3. Results

### (a) Preprint posting policies

We identified 230 eligible ecology and evolutionary biology journals, published by 69 different publishers (52, 22.6% *Wiley*; 32, 13.9% *Elsevier*; 31, 13.5% *Springer*), using the Web of Science 2023 JCR (electronic supplementary material, table S1). Across the sample, the median JIF was 2.2, and journals published a median of 36.5% of their articles as Gold OA (table 1).

**Table 1.** Journal (*n* = 230) metadata summary* based on information extracted from the Web of Science 2023 JCR. IQR, interquartile range

category	median (IQR) total citations	median (IQR) JIF	median (IQR) percentage of OA gold articles
all journals	2597 (881.8−9355.3)	2.2 (1.3−3.5)	36.5 (17.7−81.1)
journal or publisher preprint policy	3468 (1248.5−11629.3)	2.4 (1.7−3.7)	37.8 (23.1−76.2)
journal preprint policy	4068.0 (1474.0−13470.0)	2.6 (1.8−3.9)	42.0 (25.8−76.8)
publisher preprint policy	2292.0 (1163.0−10265.0)	2.2 (1.7−3.1)	31.5 (18.0−59.0)
no policy	565.0 (282.8−1021.0)	0.6 (0.5−0.9)	0.0 (0.0−97.7)

*Summary statistics for journal and publisher metadata by categories (All journals, Journal or publisher preprint policy, Journal preprint policy, Publisher preprint policy, No policy), including Median and interquartile range (IQR) of Total Citations, Journal Impact Factor (JIF) and percentage of articles published in Open Access Gold format.

Of the 230 journals, 119 (51.7%) included preprint policies in their author guidelines—either through journal-specific policies (109, 47.4%) or by directly referencing their publisher’s preprint policies (10, 4.3%). Overall, 116 (97.5%) of these 119 journals were supportive of considering preprints for publication. Three journals appeared to consider preprints on a ‘case-by-case’ basis (*Cladistics*, *Annales Zoologici Fennici* and *Trends in Ecology & Evolution*), noting that specific manuscript types (i.e. nomenclatural acts) should not be posted as preprints, that certain preprints may be permitted (e.g. those posted on *bioRxiv*) or that authors should consult the editor for guidance. However, two of these journals were published by publishers that explicitly allow preprints (*Cladistics* and *Trends in Ecology & Evolution*). A total of 73 journals (31.7%) without their own journal-level preprint policies or links to their publisher’s preprint policies were associated with publishers who were supportive of considering preprints for publication. Although no journals or publishers explicitly stated that they were not supportive of considering preprints for publication, there were 38 (16.5%) journals without any journal- or publisher-level preprint policies.

Journals with either journal- or publisher-level preprint policies had a higher median JIF (2.4, IQR = 1.7−3.7) compared with those without a preprint policy (0.6, IQR = 0.5−0.9; *p* < 0.001) (table 1).

### (b) Journal-level preprint policies and instructions for authors

Among 109 journals with journal-level policies (table 2), 89 (81.7%) outlined their preprint policies in the author guidelines section of the journal, 15 (13.8%) in various editorial or publishing policies sections and 5 (4.6%; *Annales Zoologici Fennici, Conservation Physiology, Ecosistemas, Ideas in Ecology and Evolution* and *Tropics*) in other sections of the journal (e.g. ‘terms and conditions’ or ‘submissions’). There were 79 (72.5%) journals that explicitly mentioned preprints could be posted on preprint servers, of which 15 (19.0%) specified that the servers had to be non-commercial. While 62 journals (56.9%) mentioned specific preprint servers, such as *bioRxiv*, *SSRN* and *arXiv*, only one journal (*The American Naturalist*) acknowledged *EcoEvoRxiv*—a platform tailored specifically for ecology and evolutionary biology. Nearly one-fifth of the journals (20, 18.3%) outlined other locations where preprints could be posted, including the author’s homepage and institutional repositories (electronic supplementary material, table S2).

**Table 2.** Summary of 109 ecology and evolutionary biology journals with journal-level preprint policies.

category	subcategory	journals (*n* = 109)	percentage (%)
journal-level preprint policy	explicitly allowed	106	97.2
	case-by-case determination	3	2.8
preprint policy location^a^	journal author guidelines	89	81.7
	journal policies	15	13.8
	other	5	4.6
acceptable preprint posting locations^b^	preprint servers	79	72.5
	author’s homepage	12	11.0
	institutional repositories	8	7.3
	other	23	21.1
	not specified	11	10.1
reference to specific platforms^b^	yes	62	56.9
	*BioRxiv*	*25*	*40.3*
	*SSRN*	*23*	*37.1*
	*ArXiv*	*14*	*22.6*
	*other*	*37*	*59.7*
	no	47	43.1
guidance on disclosure of preprints at time of manuscript submission	yes	24	22.0
	*cover letter*	*11*	*45.8*
	*within manuscript*	*2*	*8.3*
	*within submission portal*	*2*	*8.3*
	*not specified*	*9*	*37.5*
	no	85	78.0
guidance on timing of preprint posting relative to manuscript submission	yes	35	32.1
	*anytime*	*20*	*57.1*
	*prior to submission*	*11*	*31.4*
	*anytime prior to acceptance*	*4*	*11.4*
	no	74	69.7
guidance on linking preprint to published article	yes	48	44.0
	no	61	56.0

^a^ Preprint policy location was categorized as follows: *Journal author guidelines* covers any reference to a journal’s preprint policies within its author guidelines; *Journal policies* refer to broader editorial, publishing or ethics policy pages where the preprint policy was mentioned, and *Other* covers other locations, such as submission checklists and terms and conditions.

^b^ Percentages exceed 100% because some journals allow preprints to be posted in multiple locations (such as preprint servers, authors' homepages and institutional repositories), and on multiple servers (such as *bioRixv* and *arXiv*). Each location or server is counted separately in the analysis, meaning a single journal may be represented across multiple categories.

We identified 24 (22.0%) journals that required authors to disclose preprints at the time of manuscript submission, either in a cover letter or within their manuscript. Thirty-five (32.1%) journals (table 2) provided specific timing instructions for preprint posting relative to manuscript submission (e.g. anytime or before submission). Additionally, 35 (32.1%) journals provided an option for authors to preprint their manuscripts during the submission process using *Research Square*, *Authorea*, *SSNR*, *Advance* and *Authoring, Reviewing, Publishing, Hosting and Archiving (ARPHA) Preprints*. Three journals (2.8%; *Diversity-Basel, Ecologies* and *Fire-Switzerland*) allowed authors to upload manuscripts to their publisher’s platform following journal submission (*Preprints.org*) and three (2.8%; *Biology Letters, Journal of Evolutionary Biology* and *Proceedings of the Royal Society B-Biological Sciences*) journals supported direct integration with *bioRxiv*. One (0.9%; *Biogeosciences*) journal required all manuscripts to be posted on *EGUsphere*, facilitating public discussions alongside peer review. Fewer than half of the journals (48, 44.0%) required authors to update their preprints to include a link to the final publication. Notably, five of these journals (10.4%; *Australian Systematic Botany, Invertebrate Systematics, Pacific Conservation Biology, Rangeland Journal* and *Wildlife Research*) also required that the preprint be referenced within the Data Availability Statement of the published article.

### (c) Publisher-level preprint policies and instructions for authors

The 230 eligible ecology and evolutionary biology journals were published by 69 different publishers. Of these 69 publishers, 26 (37.7%) had a publisher-level preprint policy. All 26 of these publishers were supportive of considering preprints for publication (table 3).

**Table 3.** Summary of characteristics of 26^a^ publisher-level preprint policies that publish ecology and evolutionary biology journals.

category	subcategory	publisher preprint policies (*n* = 26)	percentage (%)
publisher-level preprint policy^a^	explicitly allowed	26	100.0
acceptable preprint posting locations^b^	preprint servers	19	70.4
	institutional repositories	10	38.5
	author’s homepage	9	34.6
	other	10	38.5
	not specified	2	7.7
reference to specific platforms^b^	yes	14	53.8
	*bioRxiv*	*8*	*57.1*
	*arXiv*	*7*	*50.0*
	*Research Square*	*3*	*21.4*
	no	12	46.2
guidance on disclosure of preprints at time of manuscript submission	yes	16	61.5
	*within manuscript*	*4*	*25.0*
	*within manuscript and cover letter*	*2*	*12.5*
	*cover letter only*	*1*	*6.2*
	*within manuscript and when submitting a manuscript to the journal (but not specified how)*	*1*	*6.2*
	*within submission portal*	*1*	*6.2*
	*disclose within the submission portal and upload a copy of the preprint during submission*	*1*	*6.2*
	*not specified*	*6*	*37.5*
	no	10/26	38.5
guidance on timing of preprint posting relative to manuscript submission	yes	16	61.5
	*anytime*	*9*	*56.2*
	*anytime prior to acceptance*	*7*	*43.8*
	*prior to submission*	*1*	*6.2*
	no	10	38.5
guidance on linking preprint to published article	yes	18	69.2
	no	8	30.8

^a^ One publisher provided different preprint guidance on acceptable posting locations (anywhere versus author’s homepage, institutional repository and non-commercial preprint servers) and timing of preprint posting relative to manuscript submission (posting anytime versus prior to acceptance).

^b^ Percentages exceed 100% because some publishers allow preprints to be posted in multiple locations (such as preprint servers, authors’' homepages, and institutional repositories), and on multiple servers (such as *bioRixv* and *arXiv*). Each location or server is counted separately in the analysis, meaning a single publisher may be represented across multiple categories.

Among publishers with a preprint policy, the majority were commercial publishers (*n* = 13), followed by not-for-profit publishers (*n* = 5; *Annual Reviews, Canadian Science Publishing, Copernicus Gesellschaft mbH, CSIRO Publishing* and *Royal Society*), university presses (*n* = 4; *Cambridge University Press, Oxford University Press, University of Chicago Press* and *University of Wisconsin Press*) and societies publishing their journals through commercial publishers (*n* = 4; *Nordic Society Oikos, Ecological Society of America, British Ecological Society* and *The Wildlife Society* via *Wiley*). Several additional society journals were also published through commercial publishers such as *Elsevier* and, in those cases, fully adopted the publisher’s overarching preprint policy. Publishers without a preprint policy represented a more diverse group, including university presses (*n* = 10), institutional publishers (*n* = 10), commercial publishers (*n* = 7), societal publishers (*n* = 6), associations (*n* = 5; *Interciencia, Natural Areas Association, Northwest Scientific Association, Asociación Española de Ecología Terrestre* and *American Association for the Advancement of Science*), not-for-profits (*n* = 2; *Resilience Alliance* and *Eagle Hill Institute*), a governmental publisher (*n* = 1; *US Fish & Wildlife Service*) and two publishers whose type could not be determined (*n* = 2; *Kamerton Publisher* and *Llc Publishing House, Kamerton*).

Of the 26 publisher-level policies, 19 (73.1%) outlined that preprints could be posted on preprint servers; four (21.1%; *Brill, Oxford University Press, Taylor & Francis* and *The Wildlife Society published through Wiley*) specified that the preprint servers had to be non-commercial. There were 14 (53.8%) publishers that referenced specific preprint servers, including *bioRxiv* (8, 57.1%) and *arXiv* (7, 50.0%) (table 3). Some publishers listed other acceptable locations for posting preprints (electronic supplementary material, table S3), such as institutional repositories (10, 38.5%) and authors’ homepages (9, 34.6%). One publisher (*Oxford University Press*) provided different instructions for authors on acceptable preprint posting locations (e.g. anywhere versus author’s homepage, institutional repository or non-commercial preprint servers) and timing of preprint posting relative to manuscript submission (e.g. anytime versus prior to acceptance), reflecting the varying member journal policies (i.e. less restrictive versus more restrictive preprint policies).

Seven (26.9%) publishers provided an option for authors to preprint their manuscripts during the submission process using *Research Square* (*Springer, Springer Nature* and *BMC*), *Authorea* (*Wiley*), *SSRN* (*Elsevier*), *Advance* (*Sage*) and *ARPHA Preprints* (*Pensoft Publishers*). Additionally, one (3.8%) publisher (*MDPI*) allowed authors to upload manuscripts to its *Preprints.org* platform after journal submission.

There were 16 (61.5%) publishers that required authors to disclose a preprint posting at manuscript submission (e.g. in the cover letter) and 16 (61.5%) that provided specific instructions on the timing of preprint posting (e.g. before submission or anytime; table 3). Eighteen (69.2%) publishers required authors to update their preprints to include a link to the final publication. Notably, one of these publisher policies (5.5%; *CSIRO Publishing*) also required that the preprint be referenced within the Data Availability Statement of the published article.

## 4. Discussion

In this evaluation of 230 ecology and evolutionary biology journals, 191 (83%) had either journal- or publisher-level policies that explicitly allowed preprints. However, fewer than half of the journals provided preprint policies in their journal-level author guidelines or offered clear instructions for authors on acceptable locations for posting preprints, timing of preprint posting relative to manuscript submission and requirements to link preprints to final published articles. These findings highlight opportunities for ecology and evolutionary biology journals, along with their publishers, to clarify and refine their preprint policies and instructions for authors to facilitate broader adoption of preprints in these fields.

We found that 83% of journals had either journal- or publisher-level preprint policies explicitly supporting preprints, which aligns with previous findings in the life sciences (90%) [27] and clinical medicine (80%) [26]. However, only half of the ecology and evolutionary biology journals provided their own journal-level preprint policies. Among publishers, fewer than 40% had a publisher-level preprint policy, aligning with findings from a 2018 study [28], which reported that less than 50% of publishers had clear preprint-related policies in place. Although some journals may indirectly adhere to publisher-level policies that support preprints, these policies may not be easily identifiable to authors when preparing their manuscripts. Authors are unlikely to search for publishers’ policies, particularly if the journal does not explicitly link to these policies within its own author guidelines or submission instructions. Furthermore, some publishers (*Wiley, Oxford University Press, University Chicago Press* and *Sage*) allow journals to establish their own preprint policies, which may differ from the publisher-level policies and instructions for authors. These publishers highlight this information on their websites and advise authors to consult individual journal pages. This level of journal-specific detail is currently not available in the Open Policy Finder, a tool designed to be used by the research community to identify publisher-level policies. As a result, authors may need to consult individual journal guidelines to ensure they have the most accurate and relevant preprint information.

Our findings suggest the need for clear and detailed journal- and publisher-level policies and instructions for authors. A previous survey of researchers found that a lack of discipline-specific guidance is a barrier to the broader adoption of preprints [31]. In ecology and evolutionary biology, nearly 70% of journal-level policies and 40% of publisher-level policies did not provide guidance on the timing of the preprint posting relative to manuscript submission. Furthermore, we found that fewer than 60% of journals outlined which preprint servers could be used by authors. In fact, only one journal endorsed *EcoEvoRxiv*, the preprint platform designed for the ecology and evolutionary biology community. Unlike other preprint services that focus on empirical, English-only content, *EcoEvoRxiv* accepts multilingual preprints, registered reports and non-traditional research article types (e.g. methods or opinion pieces) [24]. This lack of endorsement may reflect limited awareness of *EcoEvoRxiv* among journal editors and publishers, despite its field-specific features. Additionally, the absence of recommendations regarding specific preprint servers could contribute to confusion or hesitation among authors about where to share their work, potentially hindering the broader adoption of preprinting practices. Similarly, the lack of a requirement to link preprints to the final published article, as seen in more than half of the journals in our sample, may further reduce the ability to locate the preprints of published manuscripts and *vice versa*.

Journals and publishers may have reservations about the use, impact and consequences of preprints. These include the dissemination of research without peer review, the confusion that may result from having a preprinted and peer-reviewed version of the same manuscript in the literature and the impact that preprinting can have on the media attention and citations that the peer-reviewed versions of the manuscripts receive. For example, during the coronavirus disease 2019 (COVID-19) pandemic, there was an unprecedented surge in preprint use [32], with thousands of related manuscripts rapidly posted—often by authors who had never previously used preprints [33]. This raised challenges related to the spread of misinformation, particularly when unverified claims gained media traction before being debunked and retracted [32].

However, studies have consistently demonstrated that many of these concerns may not be as significant as initially thought. In particular, evidence suggests that the primary findings and conclusions reported in preprints and the corresponding final publications do not change substantially [34–36]. Although multiple versions of the manuscript represents a logistical challenge, journals could minimize any confusion by ensuring that preprints are clearly linked to their corresponding final publications. Furthermore, while media coverage of preprints has been increasing, evidence suggests that peer-reviewed articles still receive more media attention [35]. Additionally, according to multiple evaluations of preprints posted on *bioRxiv* [37,38], articles with preprints receive higher Altmetric attention scores and more citations than those without preprints. Lastly, research in clinical medicine [39] suggests that there are no significant differences in attention or citations between articles with and without preprints in the first 2 years after publication. While preprint findings should be viewed as preliminary, these previous studies should provide some reassurance to journals, publishers and other stakeholders.

### (a) Recommendations to improve preprint policies in ecology and evolutionary biology journals and publishers

Our findings suggest several opportunities for journals and publishers to improve the accessibility and clarity of their preprint policies and instructions for authors.

#### (i) Establish preprint policies

All ecology and evolutionary biology journals could aim to establish preprint policies. Journals that currently lack a preprint policy could either develop one or directly refer to their publisher’s preprint policies. Publishers that allow journals to establish their own preprint policies and instructions for authors, potentially differing from the overarching publisher’s policy, could clearly indicate this on their websites by including a statement directing authors to consult individual journal pages.

#### (ii) Standardize the location of preprint policy information

To improve preprint policy visibility, journals could standardize the location where this information is provided. While most journals with journal-level preprint policies provided this information in journal author guidelines, many journals describe their preprint policies in other journal sections, such as editorial policies, publishing policies, ethics sections, submission checklists or frequently asked questions. Consistent location across journals, ideally within the *Instructions for authors,* will make it easier for authors to locate and follow preprint policies.

#### (iii) Provide detailed guidance and examples

Journals and publishers could provide explicit and detailed guidance on preprint use in their policies and instructions for authors. This includes clearly defining where preprints may be posted (e.g. discipline-specific preprint servers such as *EcoEvoRxiv*, more general-purpose servers like *bioRxiv* or *arXiv*, institutional repositories and personal websites); when preprints may be posted (e.g. before submission to a journal or at any time); how and where authors should disclose the existence of a preprint (e.g. in cover letter or manuscript itself); and any additional required actions upon publication of the final article (e.g. linking the preprint to the final published article). Providing instructions on how to disclose preprints during manuscript submission, such as in the cover letter, can improve transparency in the review process. Additionally, providing instructions that encourage authors to reference and link the preprint to the published version of their manuscript can enhance the visibility and impact of both versions, ultimately improving accessibility to research findings. More granular preprint policies and instructions could also facilitate the systematic tracking of preprints, enabling researchers to monitor trends, identify gaps and assess the impact of preprints more effectively.

#### (iv) Facilitate integration with preprint platforms

Journals and publishers could further streamline preprint adoption by offering direct integration with preprint servers. For example, journals could offer authors the option to simultaneously post their manuscript to a preprint server during the journal submission or implement a process to allow transfer of the manuscript files and metadata from preprint servers—an approach already adopted by some journals (*Biology Letters, Journal of Evolutionary Biology* and *Proceedings of the Royal Society B-Biological Sciences)* with *bioRxiv*. Such integration could minimize the additional work for authors, eliminating the need to search for appropriate platforms and ensuring a smoother, more efficient submission process.

##### Limitations

This study has several limitations. First, we identified journals listed in the Web of Science categories of ecology and evolutionary biology and therefore did not capture additional interdisciplinary or emerging journals relevant to the field. However, the focus on these categories ensures that the selected journals represent the most commonly used ecology and evolutionary biology journals, providing a representative sample of preprint policies in these fields. Second, preprint policies and instructions may not have been identified if they were located in less accessible sections of journal websites. However, our analysis captured the majority of corresponding publisher-level preprint policies, providing a comprehensive overview of preprint policies. Third, our evaluation reflects policies at a single point in time, and the rapidly evolving landscape of preprinting and open science practices warrants continued monitoring.

## 5. Conclusion

Our systematic evaluation of journal and publisher preprint policies in ecology and evolutionary biology suggests that the majority have either journal- or publisher-level policies supportive of considering preprints for publication. Although nearly all the journals and publishers with policies support posting preprints prior to manuscript submission, fewer than half of the journals provided preprint policies in their author guidelines or offered clear instructions for authors on preprint use, including acceptable locations for posting preprints and timing of posting preprints relative to manuscript submission, creating potential barriers for authors. Our findings highlight the need for clear journal-level preprint policies that are easy for authors to identify and navigate. We call on journals to help standardize and enhance preprint policies to better support authors and foster preprint adoption across ecology and evolutionary biology.

## Data Availability

The data collected during this study and the code used for data processing and analysis are available via Open Science Framework [40]. Supplementary material is available online [41].
